# Fully automated life support: an implementation and feasibility pilot study in healthy pigs

**DOI:** 10.1186/s40635-018-0168-3

**Published:** 2018-01-16

**Authors:** Wilfried Klingert, Jörg Peter, Christian Thiel, Karolin Thiel, Wolfgang Rosenstiel, Kathrin Klingert, Christian Grasshoff, Alfred Königsrainer, Martin Schenk

**Affiliations:** 1 Department of General, Visceral and Transplant Surgery, Tübingen University Hospital, Hoppe-Seyler-Str. 3, 72076 Tübingen, Germany; 2 Department of Computer Engineering, Tübingen University, Sand 13, 72076 Tübingen, Germany; 3 Department of Anesthesiology, Tübingen University Hospital, Hoppe-Seyler-Str. 3, 72076 Tübingen, Germany

**Keywords:** Critical care, Fuzzy logic, Intensive care, Homeostasis, Closed-loop, Pigs

## Abstract

**Background:**

Automated systems are available in various application areas all over the world for the purpose of reducing workload and increasing safety. However, such support systems that would aid caregivers are still lacking in the medical sector. With respect to workload and safety, especially, the intensive care unit appears to be an important and challenging application field. Whereas many closed-loop subsystems for single applications already exist, no comprehensive system covering multiple therapeutic aspects and interactions is available yet. This paper describes a fully closed-loop intensive care therapy and presents a feasibility analysis performed in three healthy pigs over a period of 72 h each to demonstrate the technical and practical implementation of automated intensive care therapy.

**Methods:**

The study was performed in three healthy, female German Landrace pigs under general anesthesia with endotracheal intubation. An arterial and a central venous line were implemented, and a suprapubic urinary catheter was inserted. Electrolytes, glucose levels, acid-base balance, and respiratory management were completely controlled by an automated fuzzy logic system based on individual targets. Fluid management by adaption of the respective infusion rates for the individual parameters was included.

**Results:**

During the study, no manual modification of the device settings was allowed or required. Homoeostasis in all animals was kept stable during the entire observation period. All remote-controlled parameters were maintained within physiological ranges for most of the time (free arterial calcium 73%, glucose 98%, arterial base excess 89%, and etCO_2_ 98%). Subsystem interaction was analyzed.

**Conclusions:**

In the presented study, we demonstrate the feasibility of a fully closed-loop system, for which we collected high-resolution data on the interaction and response of the different subsystems. Further studies should use big data approaches to analyze and investigate the interactions between the subsystems in more detail.

## Background

Closed-loop systems exist everywhere and help making our work and life easier and safer. The need to automate procedures in the medical sector, especially in intensive care medicine, has been evident for years [1–3].

Many different studies have shown that closed-loop systems can be better and more effective than manual control [4–8]. Fuzzy logic [9] is currently one of the best means of putting closed loops into practice [10–12] as it provides the benefits of flexibility and reproducibility and can be validated as the utmost important aspect for applications in the medical sector. Following this realization and trend, more and more of these systems in the medical sector, especially for intensive care medicine, have been developed and, as shown by Bahadori et al., are well suited for workload reduction and increasing safety [13].

Nevertheless, many systems control only a single parameter. An example of such a system is the Space TGC (Space Glucose Control, B. Braun, Melsungen, Germany), which stabilizes the blood glucose level [14]. Other systems control an individual process, such as weaning [15]. There are also systems that control more complex physiological processes. An example is the LIR (learning intravenous resuscitator) algorithm by Rienhart and colleagues. Their evaluation was performed with simulations and implementation of an automated system in an in vitro as well as in an in vivo study [16–19]. Another example of a system with complex interactions is the anesthesia control. Various approaches have already been developed to control the depth of narcosis [20–22].

However, no complete closed-loop system covering various aspects of critical care is available yet. All previously described systems and many more are only subsystems that were invented to reduce workload on an intensive care unit (ICU) with a focus limited to the individual aspect of care. If such different subsystems were to be used for the same patient, interactions between them would still be unknown.

The crucial question is whether a complete system covering all aspects of therapy is possible and how the different subsystems would interact with each other. Precisely because of this lack of knowledge, we conducted a pilot study in three healthy pigs over 72 h each for the purpose of developing and integrating five subsystems and studying such a more complete closed-loop system without human interference.

## Methods

The study was approved by the local Animal Experiment Committee in accordance with the National Guidelines for Animal Care and Handling. Results for three female German Landrace pigs with a body weight of 52.1 ± 1.8 kg (mean ± SEM) are presented.

### Experiment setup

The experiment setup consists of several medical devices arranged for an ICU setting: IntelliVue MP50 (Philips Healthcare, Hamburg, Germany) for monitoring the vital parameters, Evita XL (Evita XL, Draeger Medical, Luebeck, Germany) for mechanical ventilation, Space System (B. Braun, Melsungen, Germany) for intravenous infusions, and an in-house developed electronic scale with no medical device approval to measure the collected urine’s weight which is indicated of the urine volume. Ventilation device and infusion pumps have been given remote control capabilities.

A bedside-embedded computer is used to capture and control the devices and to communicate with a central server over standard Ethernet connections. The central patient monitor and the blood gas analysis (BGA) device (ABL 800 Radiometer, Copenhagen, Denmark) are connected to the network as well.

**Fig. 1:**
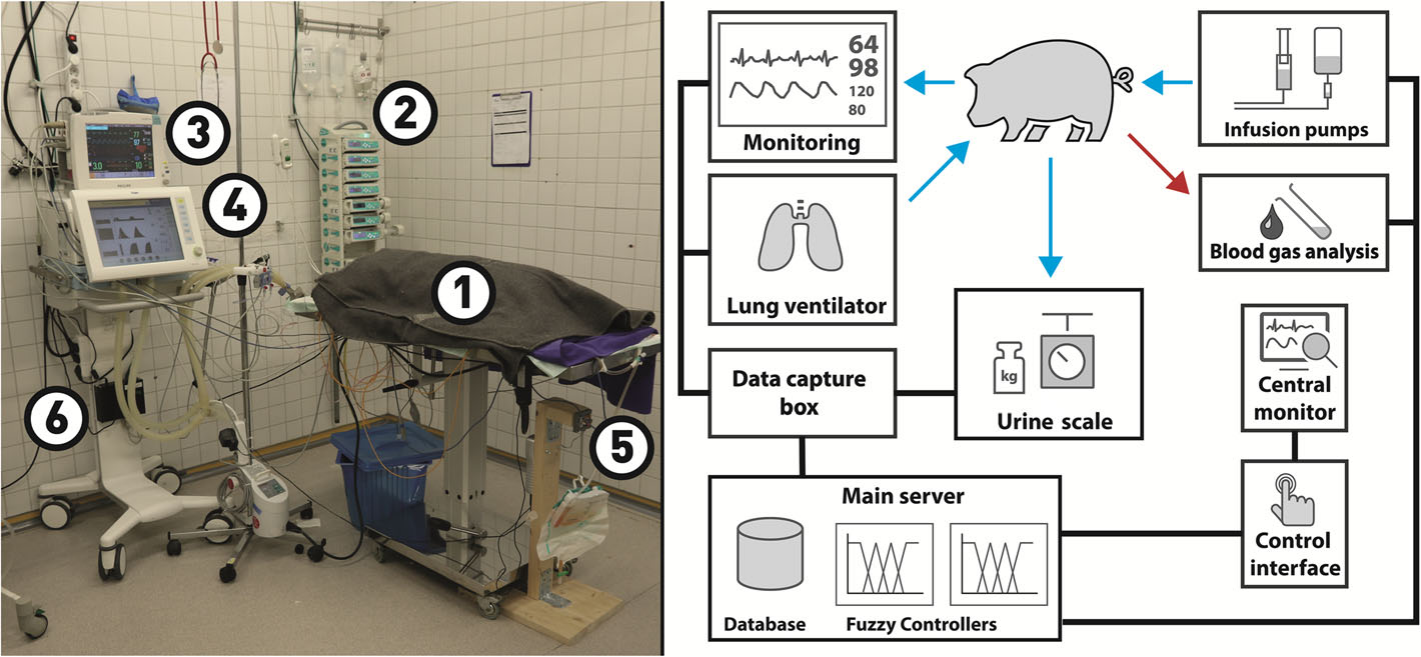
Used experiment setting (left: 1 pig, 2 infusion pumps, 3 monitoring, 4 lung ventilator, 5 urine scale, 6 data capture box) and schematic illustration (right)

Data acquisition from all used devices is performed with TICoMS (Tübinger ICU Control and Monitoring System) [23]. This framework is able to collect all data, store the measurements in a database, and handle all device connections and control commands. Figure 1 shows a photograph and a schematic representation of the used ICU setting.

### Fuzzy logic control

For this study, a controller based on fuzzy logic is used. Fuzzy logic, first introduced by Zadeh [9], uses multi-variate logic for calculation and evaluation. The fuzzy sets have two input variables: the measured value and its calculated slope. All used fuzzy sets were symmetric, and thus, no asymmetric risks were modeled.

The definition of the individual control loops for each fuzzy set is based on three user-defined parameters: the desired target value, a critical lower bound, and an upper limit, from which the membership functions with five classes (“critically low,” “low,” “normal,” “high,” “critically high”) are calculated. The defuzzified output of the fuzzy controllers is modeled as a proportional factor describing the required change from the current state of the variable to be controlled.

### Animal preparation

Animal preparation is performed as in previous studies [24–26]. In preparation for the study, the pigs were fasted overnight, given free access to water, and passed through premedication under intramuscular atropine (0.05 mg/kg) and azaperone (2–4 mg/kg) injected in a cervical neck muscle. Anesthesia is induced 10 min after premedication with intramuscular administration of midazolam (0.5–2 mg/kg) and ketamine (14 mg/kg). As soon as the pigs are asleep, a peripheral venous catheter is placed in one of the ear veins for intravenous administration of propofol (2 mg/kg). Thereafter, the trachea is intubated with a 6.5- to 7.5-F endotracheal tube, and a gastric tube is placed by a vet.

### Surgical preparations

To gain access to the arteries and veins, a five-lumen central venous catheter (ARROW DE-25855-ELOG 8.5 F × 20 cm, Arrow International Inc., Reading, PA, USA) is placed in the external jugular vein and an arterial catheter (PiCCO Catheter PV2015L20-A, PULSION Medical Systems SE, Feldkirchen, Germany) is inserted into the femoral artery. Subsequently, a suprapubic catheter is placed in the bladder. All three placements are performed with ultrasound guidance.

### Experimental protocol

Three healthy, uninjured German Landrace pigs are kept under anesthesia and treated with the help of closed-loops based on fuzzy sets for an observation time of 72 h each. The aim is to maintain homeostasis. Homeostasis is defined as all measured parameters being within physiological ranges.

The observation period commences after all catheters are inserted and all surgical and technical preparations are completed. At that point, no further human interaction, except the described maneuvers, is intended. In particular, no interaction with the controlled parameters is allowed.

During the entire experiment, anesthesia is maintained by continuous infusion of ketamine (15 mg/kg/h), fentanyl (20 μg/kg/h), and midazolam (0.9 mg/kg/h). In this first study, anesthesia was kept at a fixed level and neither manual nor automated adaptions were intended.

Ventilation is performed in a volume-controlled mode with a tidal volume of 10 mL/kg. A positive end-expiratory pressure (PEEP) of 5 mmHg is chosen to prevent atelectasis, and the FiO_2_ in the gas mixture is set at 0.40 on the medical ventilator to provide sufficient oxygen saturation.

Due to the pig’s immobilization, a low-dose thrombosis prophylaxis is performed with a bolus of 70 IU/kg of unfractionated heparin at the beginning of the observation period, and continuous infusion with initially 18 IU/kg/h unfractionated heparin is commenced following the heparin bolus. Activated clotting time (ACT) is determined every 6 h to adjust the infusion rate of unfractionated heparin to an ACT of 90–110 s.

In order to avoid preventable atelectasis and pressure marks, the pig’s position is manually changed according to a fixed scheme: the pig is positioned on its left and its right side for 8 h each, with in-between positioning on its back for 4 h. After each position change, the endotracheal tube and the trachea are manually cleared of any mucus formation by vacuum suctioning.

Electrolytes, arterial base excess (ABE), glucose, lactate, hemoglobin, and blood gases are measured with an arterial BGA every 2 h. Blood samples are collected over the arterial PiCCO catheter using a syringe overlaid with heparin. Vital parameters such as ECG, SpO_2_, temperature, arterial blood pressure, central venous pressure, and cardiac output are continuously monitored and recorded.

A volume baseline is established by continuous infusion of a balanced electrolyte solution (Jonosteril® PL 1000 ml, Fresenius Kabi Deutschland GmbH, Bad Homburg, Germany, 10 ml/kg/day). To prevent mucus formation in the respiratory tract, acetylcysteine (600 mg/l) is added to this solution. Antibiotic prophylaxis is performed by administering ceftriaxone (2 g/day).

Infusion of glucose 20% solution, calcium chloride (CaCl), sodium bicarbonate, and a second regulated balanced electrolyte solution (BES2, Jonosteril® PL 1000 ml, Fresenius Kabi Deutschland GmbH, Bad Homburg, Germany) is performed through infusion lines connected to the five-lumen central venous catheter by remote-controllable infusion pumps. The distal port (16 Ga) is used for measuring the SCVO_2_ (central venous oxygen saturation, PULSION Medical Systems SE, Feldkirchen, Germany). Through the medial one port (14 Ga) run the BES, BES2, and glucose 20% solution. The CVP (central venous pressure) and the CO (cardiac output) are measured through the medial two port (18 Ga) and the antibiotic also run over this line. TRIS buffer runs over the medial three port (18 Ga). The proximal port (18 Ga) is used for heparin. The narcotic medication is given through a Cavafix® Certo® catheter (B. Braun, Melsungen, Germany).

After the observation period of up to 72 h each, the pigs are sacrificed with an intravenous injection of embutramide (T61). Postmortem, an autopsy is performed, and tissue samples from the heart, lung, muscles, brain, kidneys, liver, and intestine are extracted and examined for change or damage.

### Controlled devices

Five automated subsystems are developed and used for fluid management and control of etCO_2_ level and the levels of glucose, calcium, and pH in the blood. These parameters are chosen because former studies showed that they had the highest variability hence the highest readjustment needs.

Glucose, calcium, and pH are managed by means of closed-loop control with fuzzy controllers. After a BGA is processed by the system, the fuzzy sets automatically adjust the infusion rates of the infusion pumps for glucose 20% solution (G20), calcium chloride, and sodium bicarbonate.

Also, the etCO_2_ is regulated with a fuzzy controller, adapting the respiratory rate based on a 5-min average reading of the etCO_2_ level measured at the ventilator.

For control of the intravascular volume, an infusion pump with a BES2 is used. Adaption is based on a volume need analysis (VNA). This analysis is a ventilation maneuver based on the Pmcf method (mean circulatory filling pressure, PMS) [27].

To perform this maneuver, PEEP is automatically set to the peak inspiratory pressure (PIP) level for 20 s by remote control at the ventilation device. During these 20 s and the subsequent 60 s, systolic arterial blood pressure is monitored. From these measurements, VNA delta is automatically calculated as the difference between maximal and minimal arterial pressure. This maneuver is repeated regularly with a variable interval, depending on the calculated volume status.

The resulting VNA delta is processed using the simple if/then/else rule to distinguish volume need, and the BES2 infusion rate was adapted accordingly. If the difference is less than the defined target threshold value, no additional volume is needed and the next VNA delta is measured 60 min later. If the VNA delta is equal to or larger than the threshold, additional volume is given. In this case, a bolus of 2 ml/kg is administered and the next VNA delta is measured after 15 min.

## Results

Evaluation of the automated system with multiple closed-loop subsystems was performed successfully in three pigs observed for 72 h each. In general, the various vital parameters were stable in all pigs during the whole observation period. No human interaction beyond the described protocol was needed, especially not for the automatically controlled parameters.

**Fig. 2:**
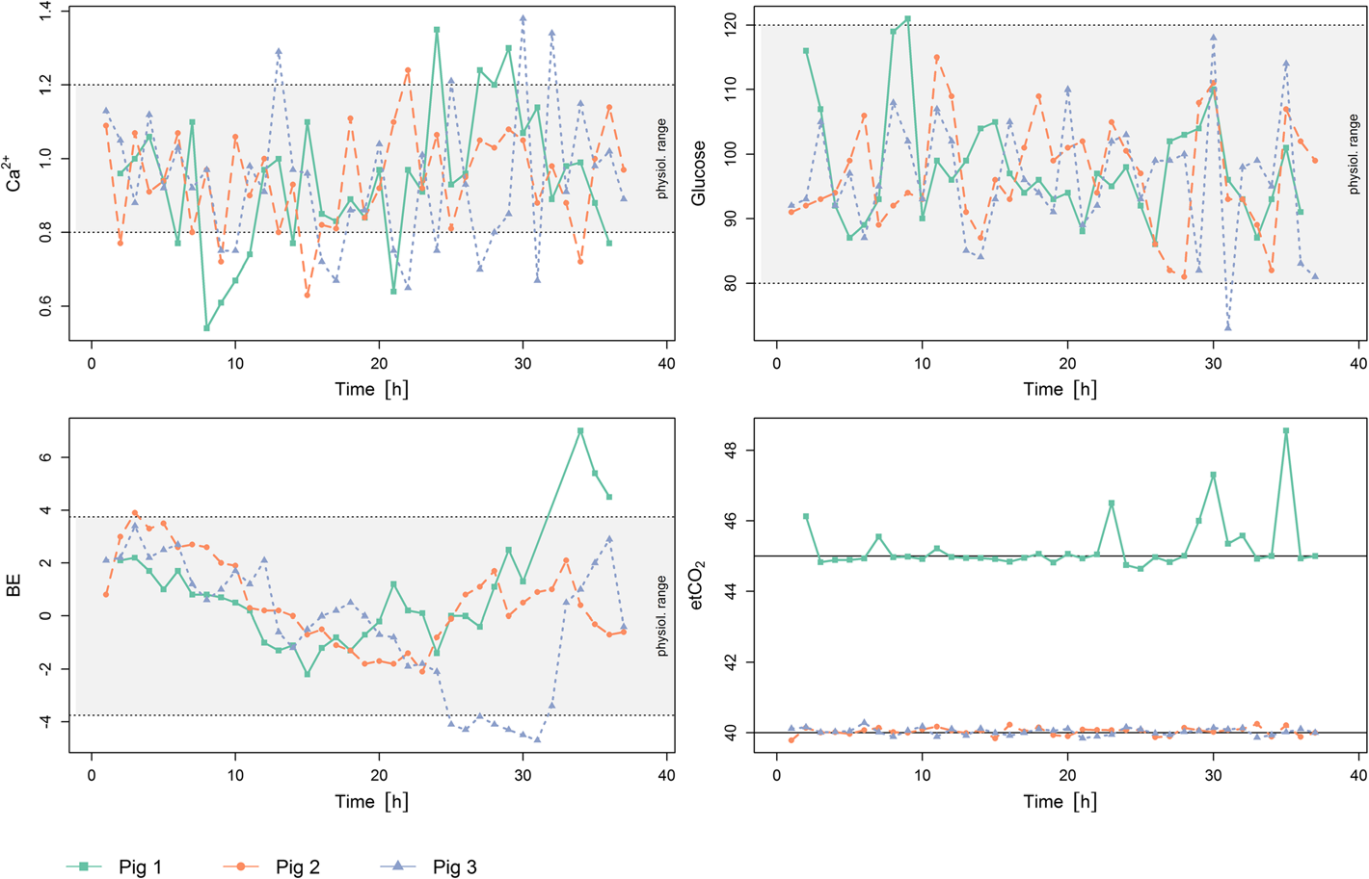
Results for the fuzzy-controlled parameters and set targets (horizontal lines) for the individual pigs: calcium levels (top left), glucose levels (top right), BE level (bottom left), and etCO_2_ (bottom right)

In all three healthy, unstressed pigs under general anesthesia, free arterial calcium measurements were within the physiological range 73% of the time, with the limits being exceeded only sporadically. The measured arterial blood glucose level was almost always (98%) within the defined physiological limits, and the ABE was kept within physiological ranges in 89% of the performed measurements. However, some outliers for individual measurements can be seen in the observation of all three pigs. In 98% of the measurements, etCO_2_ was within the physiological range fluctuating around the set targets. For the first pig, a target etCO_2_ of 45 mmHg was chosen, whereas in the other two pigs, this target was set at 40 mmHg. All measurements for the fuzzy-controlled targets are shown in Fig. 2. Whether similar results will be obtained from critically ill pigs over a longer time or from clinical application in humans must be investigated in secondary studies.

**Fig. 3:**
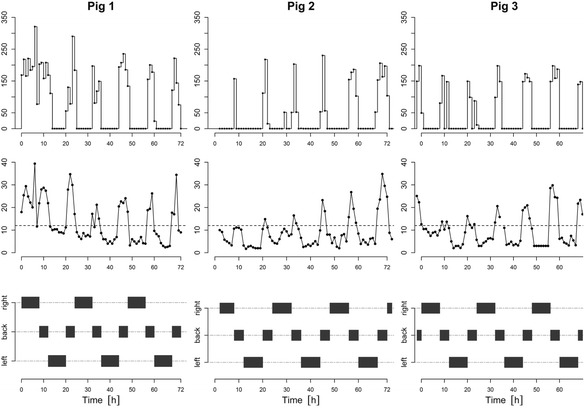
Results for the volume need in relation to the pigs’ bedding position for the three observed pigs from left to right. Rate of the second balanced electrolyte solution (BES2) as mean of the boluses (top), mean value of the measured VNA delta (middle), and position of the pig on the operating table (bottom)

The average infusion rates of the BES2 as calculated by the algorithm, the VNA deltas, and the current position of the pigs are shown in Fig. 3. It can easily be observed that especially in the dorsal position, the VNA deltas were very high, whereas when the pigs were bedded on their left or right side, no additional volume was needed for most of the time.

The autopsy performed after the observation period showed no visible anomalies and a maximum of 20 ml ascites. The tissue samples collected from the heart, lung, muscles, brain, kidney, liver, and intestine were analyzed and examined histologically. There, too, no malformations or changes were observed.

## Discussion

Automated electronic systems are recommended for the purpose of gathering data and generating predictions in the medical sector [28], and integral systems often offer a more accurate, less time-consuming, and more cost-effective process [29]. Intensive care platforms require timely processing of data retrievals to guarantee the continuous display of recent patient data, and automatic control loops are beneficial for optimizing data processing in the ICU [30]. As more and more data from different sources are integrated into a central system, therapeutic closed-loop systems utilizing data from various sources and interacting with different devices can be considered. However, effects and interactions of such systems need to be evaluated. With this study, we are the first to present a fully closed-loop therapeutic system with various subsystems working simultaneously and provide first insights into their interactions that need to be considered.

Another important aspect in the process of automation is the role of caregivers. Miller et al. [2] state that closed-loop systems are not intended to replace the anesthesia provider. Our system is designed in this spirit by reducing workload through automation of regulatory loops and therefore providing more time to think about the therapeutic targets and the overall picture as this is one of the important aspects caregivers should be able to focus on. Therefore, human interaction with therapy was neither allowed nor necessary during the automated phase of our study, which allowed us to observe coherences and interactions. This aspect may become increasingly important if automated systems are established and caregivers play a supervising instead of an acting role with their knowledge and human responsiveness still being of importance, especially in the case of rapidly evolving clinical conditions and technical failures. Our study employed only healthy pigs, and there is still a long way to go before a closed-loop system in the ICU can provide care for seriously ill patients undergoing severe pathophysiological changes. Nonetheless, using only such relatively simple control systems, we were able to provide an essential next step toward those goals by showing feasibility and observing some important interactions between the subsystems that need to be studied more profoundly and be accounted for in further research.

Closed-loop systems have been demonstrated to perform better and be more effective than manual control in the clinical sector [4–8]. Until now, a fully integral system with multiple subsystems acting simultaneously was only a theoretical construct. The first steps toward realizing a fully closed-loop system were made by various researchers who selected individual tasks and automated them [14, 15, 31, 32]. We went a step further and were able to show for the first time that a fully integral system consisting of multiple closed-loop controls is feasible.

Regarding the choice to use fuzzy controllers in comparison to other methods of control, Doyle et al. [33] enumerated design objectives, variables, and challenges involved in the development of an artificial pancreas (AP) and examined three different types of glucose management with insulin pumps using model predictive control (MPD), proportional integral derivative (PID), and fuzzy logic. During their studies, in which only the first two controller types were evaluated in depth, glucose was kept within the euglycemic range on average 71% of the time, showing the potential and benefits of such automated closed-loop systems. The fuzzy logic control developed by us for glucose management in our setting was able to keep glucose within the physiological range for 98% of the measurements, substantiating the benefit of automated management by means of closed-loop systems.

Although we used only simple fuzzy logic rules for the closed loops, the pigs’ conditions were kept stable for the whole observation period without showing any provable signs of harm after death. Especially, the etCO_2_ levels of pigs 2 and 3 were successfully stabilized. This was facilitated by the high temporal resolution of the measured values from the ventilator and the chosen adaption interval of 5 min, providing a stable closed-loop system where no over- or undershooting of the target etCO_2_ level occurred. This might not be the case with critically ill pigs or sick humans which could be vigorously manipulated during critical care. Such manipulating during their stay in the ICU can change physiological inputs and could lead to much less desirable performance of the closed-loop system. Further studies must be performed to proof the closed-loop system’s stability. Furthermore, our regulated parameters controlled by the well-established system must be replenished with additional parameters like vasopressors, narcosis, and supplementary electrolyte (potassium).

Rose, Liu, and Burns et al. [34–36] proposed that an automated closed-loop system may improve adaptation of mechanical support for a patient’s ventilator needs and facilitate systemic and early recognition of the patient’s ability to breathe spontaneously. As we were able to successfully control etCO_2_ level and faced no respiratory problems, we succeeded in showing that such an integral system is not harmful for pigs. Rose et al. [36] furthermore stated that automated closed-loop systems may result in reduced duration of weaning, ventilation, and ICU stay. This should be examined in further clinical studies as our current study provides only a first but essential step toward closed-loop management of ventilation needs.

As shown regarding ventilation needs, an important aspect of every step toward automation is the availability of data with high temporal resolution. If the required parameters are collected at a reduced rate, regulation will become more complicated. Some examples of this circumstance are the controls performed for glucose, and ABE as the adaptation rate is limited by the rate of BGA measurement. Due to this restricted knowledge, we observed some outliers in our study that may have been detected with a delay, thus requiring significant amounts of time for the control system to counteract. Despite this measurement delay, the reaction time of our fuzzy controllers was still sufficient and thus caused no harm to the pigs. Our aim for future studies is to achieve faster closed loops with shorter response times to reduce such outliers. However, not only temporal changes play an important role but also other aspects like the kinetics of the interacting systems facilitate those changes and need to be considered. We therefore aim to correlate the frequency of arterial BGA measurement and the change rate of the monitored parameters like glucose or ABE and replace parameters with surrogates available at higher frequencies. One need not look far to see that special situations like septic scenarios require more BGAs within a shorter time frame than are collected for stable patients or healthy pigs like those examined in this study. Nevertheless, making the control dependent solely on infrequently performed manual measurements may not be sufficient.

We considered only simple correlations and interactions for developing the fuzzy sets and their regulations. However, our results taught us that there are many interactions between the different parameters that should be considered. Random outliers without any reason due to measurement could be seen, but systemic outliers due to failing system could not be seen in our study.

**Fig. 4:**
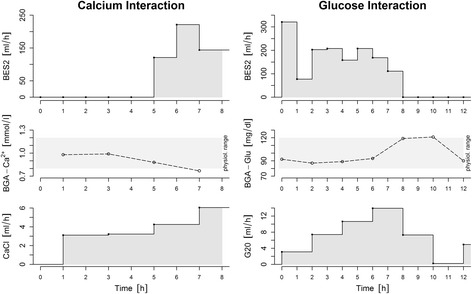
Interactions between controlled parameters and variable BSE2 infusion for calcium (left) and glucose (right) with infusion rate, BGA measurement, and corresponding BSE2 infusion shown from top to bottom

For example, in the used control system for calcium, it became obvious that the measured blood level is not affected only by the infusion rate of the CaCl pump, but by the other infusions and other systemic interactions, too. Figure 4 shows a short time frame for the observed calcium level in a pig. After increasing the BES2 rate, the calcium level drops and the calcium control system counteracts only at a significantly late time. These calcium outliers are a reaction of the interaction.

A similar observation can also be made for control of the glucose level shown in Fig. 4. After significant reduction of the BES2 rate by the volume control subsystem, the concentration of glucose is increased due to the now relatively less diluted administration of G20.

These guiding observations show that the various controlling systems must be able to communicate with each other and react to changes in other systems in order to be able to counter such effects. Future developments should therefore implement communication channels between the subsystems and incorporate the interactions. Therefore, the rule-based fuzzy logic set allows for the integration of different additional parameters to calculate a specific target value.

An important restriction worth mentioning is, of course, the animal study itself as such experimental results cannot be completely applied to humans. Additionally, the study was conducted only on healthy pigs without any co-morbidity like sepsis or intracranial pressure. With regard to the apparently low number of three animal subjects for this proof-of-concept study, the total observation time equivalent to 216 h of intensive care life support should be considered instead. In terms of fluid management, we were able to show that VNA delta varies depending on the pig’s bedding position, especially when lying on their backs. This knowledge provides a good foundation for subsequent studies that could be designed to determine whether and evaluate how pigs have different volume requirements depending on bedding and other physiological factors, species, and their potential applicability to human patients.

## Conclusions

We established and evaluated a fully closed-loop therapeutic system with various differently subsystems working simultaneously and presented first insights into their interactions. Our study was conducted with three healthy German Landrace pigs as a baseline for research on critically ill pigs and humans. We proofed the feasibility of our developed system and observed some important interactions which should be examined more preciously in further studies.

## Abbreviations

ABE: Arterial base excess
ACT: Activated clotting time
BES2: Second balanced electrolyte solution
BGA: Blood gas analysis
CaCl: Calcium chloride
CO: Cardiac output
CVP: Central venous pressure
G20: Glucose 20% solution
ICU: Intensive care unit
PEEP: Positive end-expiratory pressure
PIP: Peak inspiratory pressure
SCVO_2_: Central venous oxygen saturation
TICoMS: Tübinger ICU Control and Monitoring System
VNA: Volume need analysis
